# Oral Zinc Sulfate for Prevention and Treatment of Chemotherapy-Induced Oral Mucositis: A Meta-Analysis of Five Randomized Controlled Trials

**DOI:** 10.3389/fonc.2018.00484

**Published:** 2018-11-19

**Authors:** Xu Tian, Xiao-Ling Liu, Yuan-Ping Pi, Hui Chen, Wei-Qing Chen

**Affiliations:** ^1^Chongqing Key Laboratory of Translational Research for Cancer Metastasis and Individualized Treatment, Department of Gastroenterology, Chongqing Cancer Institute, Chongqing University Cancer Hospital, Chongqing, China; ^2^Editorial Office, TMR Integrative Nursing, TMR Publishing Group, Tianjin, China; ^3^Key Laboratory for Biorheological Science and Technology of Ministry of Education, Department of Nursing, Chongqing Cancer Institute, Chongqing University Cancer Hospital, Chongqing, China

**Keywords:** chemotherapy, oral mucositis, zinc sulfate, systematic review, meta-analysis

## Abstract

Chemotherapy-induced oral mucositis is an extremely serious complication faced by cancer patients. The role of oral zinc sulfate in preventing and treating chemotherapy-induced oral mucositis remains a subject of debate. The aim of this systematic review is to assess the potential of oral zinc sulfate to alleviate this morbid condition. A systematic search was conducted electronically in PubMed, EMBASE, and the Cochrane Central Register of Controlled Trials (CENTRAL) to capture all potential randomized controlled trials investigating efficacy and safety of oral zinc sulfate in prevention and treatment of chemotherapy-induced oral mucositis, and the retrieval time was limited from their inception to April 2018. We assigned two independent investigators to perform a search, screen citations, extract information, and evaluate the risk of bias in all included trials. Subsequently, the RevMan 5.3 software was utilized to perform all statistical analyses. We included five eligible studies involving 352 patients. Meta-analysis based on limited data revealed that oral zinc sulfate failed to decrease the incidence of chemotherapy-induced oral mucositis (RR [relative risk] = 0.52, 95% CI [confidence interval] = 0.17–1.64) as well as relieve chemotherapy-induced oral mucositits grade (RR = 0.62, 95% CI = 0.11–3.56; RR = 0.70, 95% CI = 0.29–1.71). Moreover, qualitative analyses also suggested that oral zinc sulfate was not associated with reduced oral pain intensity, delayed onset of chemotherapy-induced oral mucositis, decreased adverse events, or improved quality of life compared with control regimes. Oral zinc sulfate may not provide clinical benefits in preventing or reducing incidence, severity, or pain intensity of chemotherapy-induced oral mucositis in cancer patients. However, more studies with large-scale and rigorous methods are warranted for the purpose of further investigating efficacy and safety of oral zinc sulfate for this pathologic condition due to the presence of limitations.

## Introduction

Oral mucositis is a common morbid condition among cancer patients undergoing chemotherapy or radiotherapy or concurrent chemoradiotherapy. In fact, oral mucositis is currently deemed as a significant result of a series of inflammatory changes occurring in oral mucosal epithelial cells due to cytotoxic effects of these anticancer therapies (1, 2). Previous studies reported that 81.3–90% of patients with cancer (3) or acute leukemia (4) who were treated with chemotherapy suffered from oral mucositis. Cancer patients who are diagnosed definitively with chemotherapy-induced oral mucositis will experience pain, physical constraints, and psychological discomfort (4). Moreover, chemotherapy-induced oral mucositis made a negative impact on nutritional intake, mouth care, and the quality of life (QoL) of cancer patients, and it also imparted substantial economic costs on individuals and society (2, 4–6).

Because the incidence of chemotherapy-induced oral mucositis among cancer patients and corresponding consequences are extremely serious, several prophylaxes and treatments have been explored (6, 7); however, effects of those interventions, such as low-level laser therapy and several organic products (8), have not yet been completely confirmed (6). Recent evidence suggests that zinc has the capability of enhancing gastrointestinal epithelial barrier function and consequently decreasing cell death and detachment (9). Several studies have been performed to investigate the role of oral zinc sulfate in chemotherapy-induced oral mucositis (6, 10–13). Of these studies, four (6, 10, 11, 13) supported the idea that zinc has the potential of reducing incidence and severity of chemotherapy-induced oral mucositis in cancer patients. However, Mansouri et al. (12) argued that there was no benefit when oral zinc sulfate was prescribed for the treatment of high-dose chemotherapy-induced oral mucositis. Thus, efficacy and safety of oral zinc sulfate for chemotherapy-induced oral mucositis remain controversial. It must be noted, however, that it is essential to investigate separately the efficacy of oral zinc sulfate in the prevention and treatment of chemotherapy-induced oral mucositis because differences between radiotherapy and chemotherapy cannot be ignored (14). We consequently performed this systematic review and meta-analysis to comprehensively assess the efficacy and safety of zinc sulfate for chemotherapy-induced oral mucositis.

## Methods

This systematic review was designed according to recommendations released by the Cochrane Collaboration (15). We reported all results in accordance with the criteria listed in preferred reporting items for systematic review and meta-analysis (PRISMA) (16). As all statistical analyses in this study were performed based on previous studies, informed consent was not required. We also registered the protocol of our systematic review at the International Prospective Register of Systematic Reviews (PROSPERO), and a unique identifier of CRD42018093605 has been approved. Moreover, the full-text protocol can be obtained from Medicine (17).

### Selection criteria

We designed the following inclusion criteria to capture any eligible studies: (i) all adult patients received chemotherapy, regardless of the dose of chemotherapy; (ii) patients assigned to the study group were administered with zinc sulfate orally, and ones enrolled in the control group were instructed to take placebo or other active drugs, which were similar to zinc sulfate in shape, taste, and color; (iii) incidence of chemotherapy-induced oral mucositis and chemotherapy-induced oral mucositis grade were regarded as the primary outcome, and oral pain intensity, onset of chemotherapy-induced oral mucositis, zinc-related adverse events (AEs), and the QoL were defined as the secondary outcome; (iv) only randomized controlled trials (RCT) were considered, however, an abstract with sufficient information would also be considered; and (vi) only studies published in English or Chinese were viewed as candidates because a translator who is well versed in other languages was not assigned.

We excluded a study if it covered one or more of the following criteria: (i) lack of essential information; (ii) duplication with poor methodology and insufficient data; and (iii) studies in which patients received radiotherapy or chemoradiotherapy.

### Definition of outcomes

In this study, we defined the incidence of chemotherapy-induced oral mucositis as the value of the number of patients who experienced chemotherapy-induced oral mucositis with all-grade divided by the total number of cancer patients who completed this study. We graded the severity of chemotherapy-induced oral mucositis based on World Health Organization (WHO) criteria (18) or the Spijkervet scale (19). We graded chemotherapy-induced oral pain intensity according to the visual analog scale (VAS) (6). The onset of chemotherapy-induced oral mucositis was viewed as the time when oral mucositis was definitively diagnosed. Zinc-related AE was regarded as any AE that occurred after taking zinc sulfate orally. The QoL must be assessed by using validated scales in individual studies, for example the European Organization for Research and Treatment of Cancer (EORTC) LQ-OES18 (10).

### Identification of citations

We electronically searched all potential citations in PubMed, the Cochrane Central Register of Controlled Trials (CENTRAL), and EMBASE, and the time range was limited from their inception to April 2018. We used the following terms including “zinc,” “oral mucositis,” and “random” to construct all search strings. We summarized all search algorithms in Supplemental Digital Content 1. We also manually checked the bibliographies of all included studies and topic-related reviews to capture any potential studies. Moreover, we searched Clinicaltrial. gov so that any ongoing trials reporting sufficient data were identified. In the current stage, we addressed all disagreements by consulting a third senior reviewer.

### Data extraction

We used a data extraction sheet (available in Supplemental Digital Content 2), which was used in our previous study (20), to abstract essential information. Firstly, we imported all captured items into the EndNote software V.X7. Secondly, we assigned two investigators to extract the basic characteristics of all eligible studies and data for each outcome including leading author, publication year, age of participants, sample size, details of intervention regimes, and outcomes of interest. The corresponding author would be contacted if sufficient data could not be extracted from eligible studies.

### Quality assessment of eligible individual studies

We assigned two independent investigators to appraise the risk of bias of each trial by using the Cochrane risk of bias assessment tool (21). Each individual study was assessed based on six domains (15): randomization, allocation, blind, incomplete data, selectively reported, and other bias. After appraising the risk of bias, individual studies were labled as “low risk of bias,” “unclear risk of bias,” or “high risk of bias” (15). A third investigator was consulted to solve inconsistent results concerning the judgment on the risk of bias in each study. Finally, we graded the overall quality of all eligible studies as moderate if most of the eligible studies were labeled as unclear or low risk of bias.

### Statistical analysis

We selected the random-effect model, which considered within and between studies heterogeneity simultaneously, to perform all statistical analyses (22). For dichotomous data, we selected risk ratio (RR) and 95% confidence intervals (CIs) to express summarized estimates, and for continuous data, we selected mean difference (MD) and 95% CIs to express pooled estimates (15). We tested the heterogeneity among eligible studies according to the following steps: we firstly described the heterogeneity based on the Chi square test (i.e., Cochrane Q) (23), and then we selected I^2^ statistic to quantitatively estimate the proportion of the overall variation that is attributable to between study heterogeneity (24). If I^2^ statistic was ≤ 50%, we considered these studies as heterogeneous, and if they were = 50%, then we judged them to be homogeneous (24). To detect the publication bias, we drew a funnel plot for a single outcome with more than 10 eligible studies (25, 26). For studies with multiple-arm design, essential data were extracted based on methods recommended by the Cochrane Collaboration (15). If we extracted the median, range, and sample size from eligible studies, then we would estimate the mean and variance (standard deviation, SD) according to the method proposed by Hozo et al. (27). Finally, all statistical analyses were completed using the RevMan software version 5.3 (Copenhagen, Denmark: The Nordic Cochrane Centre, The Cochrane Collaboration, 2013) (15).

## Results

### Identification and selection of studies

**Figure 1:**
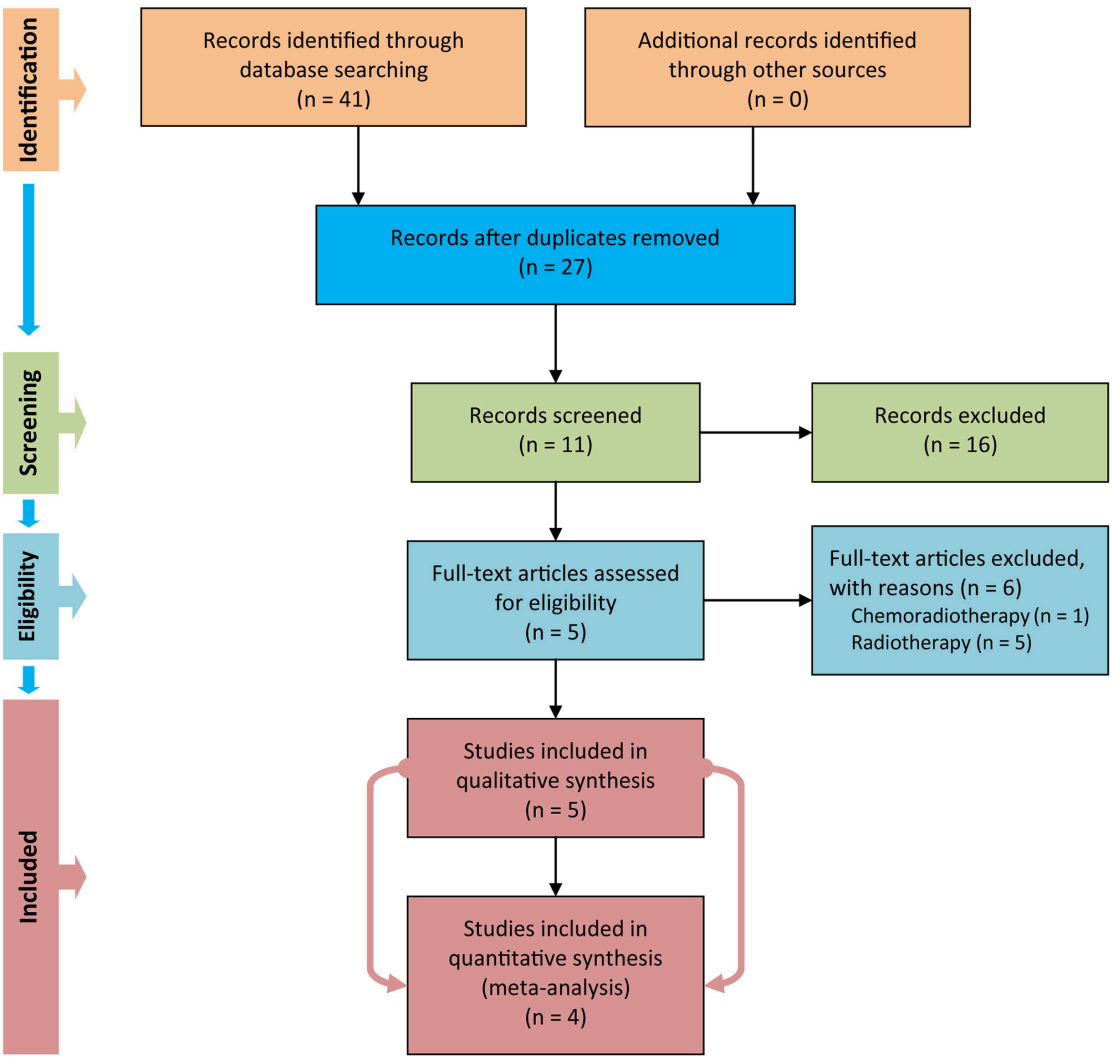
Flow diagram of retrieval and screen of study.

We used Figure 1 to delineate the steps of retrieving and selecting studies. We initially captured 41 records in PubMed, EMBASE, and CENTRAL databases after performing the electronic search. We designed a three-step approach to screen and select eligible studies. **Firstly, we removed duplicate records by using the duplicates removal function embedded in the EndNote software**. Secondly, we excluded 16 records after screening title and abstract of the remaining records. Thirdly, we eliminated 6 studies after carefully checking the full text of the remaining 11 studies because of the following reasons: in one study (28), patients were instructed to be assigned to the study group to receive chemoradiotherapy, and in five studies (29–33), patients were mixed with the radiotherapy regime. We thus included five eligible trials (6, 10–12, 34) to perform qualitative synthesis. However, only four eligible studies (6, 10–12) were in the end eligible for quantitative analysis, because one study (34) failed to report valid data.

### Characteristics of eligible studies

We prepared Table 1 to document the basic characteristics of all five eligible studies. All articles (6, 10–12, 34) were published between 2011 and 2018 and performed in Iran. In these trials, 352 patients were recruited, and sample size in a single trial ranged from 30 to 140 with a median of 60. In all these studies, four (10–12, 34) were of double-blind design and the remaining one (6) was of triple-blind design. One study (12) designed a high-dose chemotherapy regime. Four studies (6, 10–12) used WHO criteria to assess the severity of oral mucositis, and one study (34) adopted the Spijkervet scale to assess it. All studies reported a comparative baseline for all recruited participants.

**Table 1:** Basic characteristics of all five eligible studies.

**Study**	**Country**	**Sample size**		**Sex (male/female)**		**Age (yrs) (SG vs. CG)**	**Intervention regime**		**Outcomes**
		**SG**	**CG**	**SG**	**CG**		**SG**	**CG**	
Mehdipour et al. (13)	Iran	15	15	n.r.	n.r.	n.r.	10 ml of 0.2% zinc sulfate mouthwash two times per day for 14 days	10 ml of 0.2% chlorhexidine mouthwash two times per day for 14 days	Mucositis severity
Arbabi-kalati et al. (10)	Iran	25	25	13/12	13/12	18–70 vs. 18–79	220 mg zinc sulfate capsules daily until the end of chemotherapy treatment	three placebo capsules that were similar in shape, taste, and color to the zinc sulfate capsules	Incidence of oral mucositis, mucositis severity, degree of pain, quality of life
Mansouri et al. (12)	Iran	30	30	22/8	18/12	30.87 vs. 27.13	220 mg zinc sulfate capsule twice a day with 12 h interval	placebo twice a day with 12 h interval	Incidence of oral mucositis, mucositis severity, onset of oral mucositis
Gholizadeh et al. (11)	Iran	70	70	25/45	29/41	46.3 ± 12.6 vs. 49.4 ± 11	220 mg zinc sulfate capsules three times daily	placebo capsules that were similar in shape, taste, and color to the zinc sulfate capsules	Incidence of oral mucositis, mucositis severity, degree of pain
Rambod et al. (6)	Iran	36	36	23/18	24/18	39.65 ± 17.07 vs. 33.80 ± 13.73	42 (150 mg) zinc three times daily for 14 days from the first day of chemotherapy	42 placebo three times daily for 14 days from the first day of chemotherapy	Incidence of oral mucositis, onset of oral mucositis, severity of oral pain

*SG, study group; CG, control group; yrs, years*.

### Risk of bias of eligible studies

**Figure 2:**
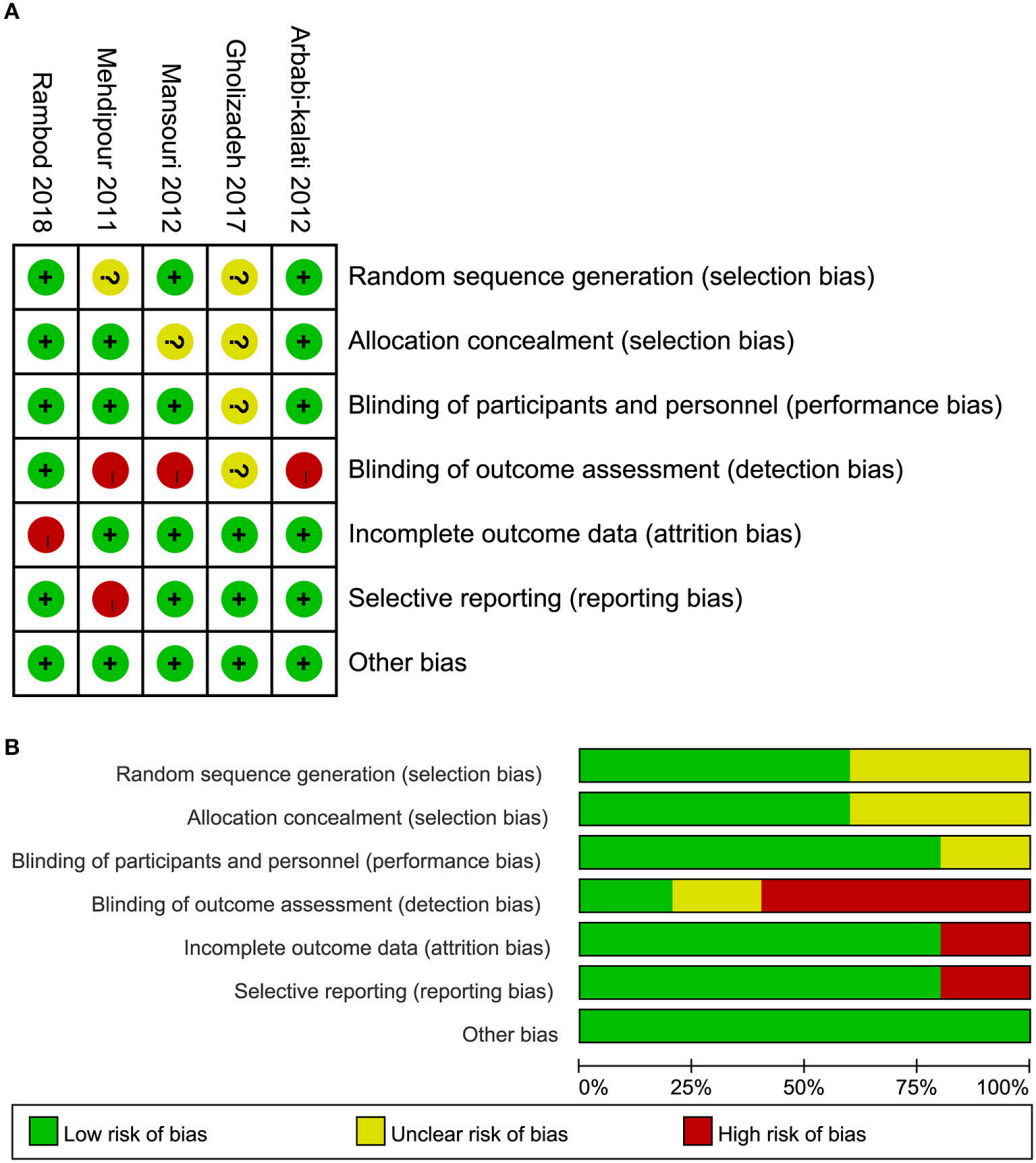
Risk of bias of all five eligible studies. **(A)** Risk of bias summary and **(B)** risk of bias graph.

Of these five eligible studies, three (6, 10, 12) adopted appropriate methods, such as an online statistical randomization program and randomized block in Microsoft Excel 2007 to generate random sequencing, three (6, 10, 34) appropriately assigned the patients into each group, and four (6, 10, 12, 34) correctly blinded researchers and/or patients. It is to be noted, however, that two studies (6, 34) were judged as high risk of bias because of incomplete data and selective reporting, respectively. In general, the overall quality of all eligible trials was moderate. The risk of bias of a single study and the overview of all studies are depicted in Figure 2.

### Incidence of oral mucositis

**Figure 3:**
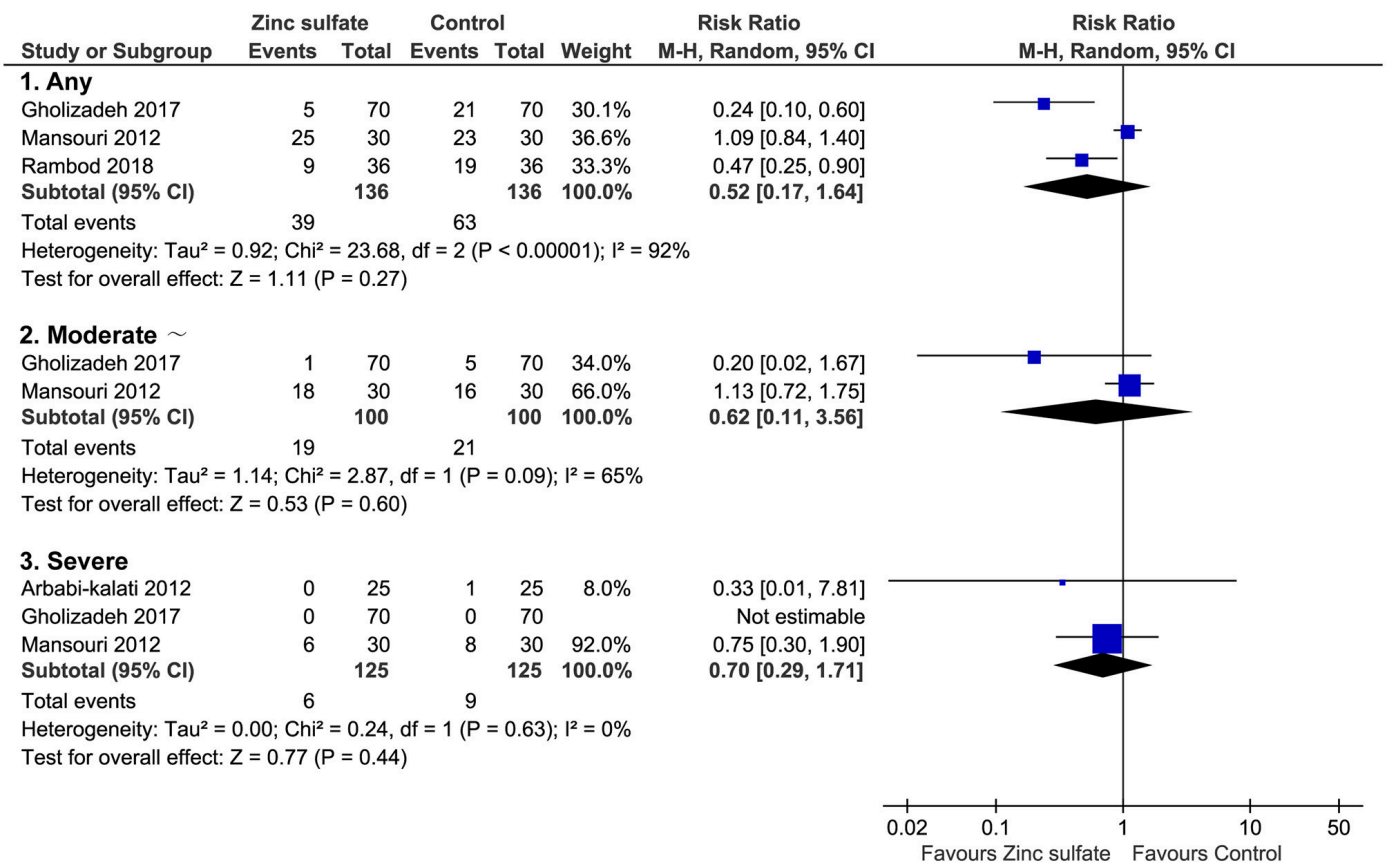
Meta-analysis of incidence of oral mucositis. Figure shows the incidence of oral mucositis irrespective of severity of oral mucositis, moderate and severe oral mucositis, and severe oral mucositis alone. CI, confidence interval; M-H, Mantel-Haenszel.

Of these five included trials, three (6, 11, 12) covering 272 patients reported the incidence of oral mucositis after the patients were instructed to take zinc sulfate orally. Meta-analysis revealed that the difference in the incidence of oral mucositis between zinc sulfate and control groups was not significant (three RCTs; RR = 0.52, 95% CI = 0.17–1.64; *P* = 0.27; I^2^ = 92%; Figure 3A).

### Severity of oral mucositis

Of all eligible studies, three (10–12) provided specific numerical data on the severity of oral mucositis, which can be used to perform meta-analysis. Numerical data on moderate and severe oral mucositis was reported in two studies (11, 12), and the pooled result suggested that the difference between the two groups was not statistically significant (two RCTs; RR = 0.62, 95% CI = 0.11–3.56; *P* = 0.60; I^2^ = 65%; Figure 3B). Three studies reported severe oral mucositis, and meta-analysis revealed that the difference in this given outcome between the two groups was not statistically significant (three RCTs; RR = 0.70, 95% CI = 0.29–1.71; *P* = 0.44; I^2^ = 0%; Figure 3C).

Moreover, one study (34) also reported the incidence of oral mucositis, however, numerical data, which can be used in meta-analysis, could not be obtained. Thus, we adopted a descriptive analysis method to summarize the findings. Mehdipour et al. (34) designed a double-blind randomized trial, and 30 patients were randomly divided to receive either zinc sulfate or chlorhexidine gluconate. They found that mean severity scores were generally lower in zinc groups compared with controls at all four time intervals evaluated, but only differences in weeks 2 and 3 were statistically significant (*P* = 0.025).

### Severity of oral pain

Of all five eligible studies, two (10, 11) considered the severity of oral pain as an outcome. Arbabi-kalati et al. (10) found that pain intensities from the third visit (chemotherapy week 6) until the tenth meeting (chemotherapy week 20) exhibited statistically significant differences between drug and placebo groups, which indicated that pain intensity in the drug group was less than that in the placebo group (*P* < 0.005). Gholizadeh et al. (11) performed a randomized trial including 140 acute myeloid leukemia (AML) inpatients to investigate whether zinc prevents oral mucositis associated with chemotherapy and showed that zinc supplementation was not associated with decreased pain intensity, however the placebo's efficacy for the relief of oral pain was significantly larger in the control group than that in the zinc group at the end of the 4th week of treatment (*P* = 0.03).

### Onset of oral mucositis

One eligible study (6) investigated the onset of oral mucositis and showed that the onset of mucositis occurred on days 5.83 (standard deviation [SD] = 3.37) and 4.58 (SD = 2.47) in the experimental and control groups, respectively. However, results of an independent *t*-test indicated no significant difference between the two groups regarding the onset of mucositis (*t* = −0.95, *p* = 0.34).

### Zinc related adverse events

For all five eligible studies, Mansouri et al. (12) enrolled 60 patients undergoing high-dose chemotherapy to explore the role of oral zinc sulfate in the prevention of chemotherapy-induced oral mucositis in a double-blind, randomized, placebo-controlled trial and failed to find any significant unwanted side effects in the zinc sulfate group as compared with the placebo group.

### Quality of life

In all these eligible studies, Arbabi-kalati et al. (10) regarded the QoL as the outcome. These authors used the EORTC QLQ-OES18 questionnaire to evaluate the QoL and found that the difference in scores of the EORTC QLQ-OES18 questionnaire was not statistically significant between zinc and placebo groups (*P*-value ranged from 0.15 to 0.91).

### Publication bias

In this systematic review and meta-analysis, we defined six outcomes. However, only incidence of oral mucositis and severity of oral mucositis met the criteria of performing meta-analysis. It should be noted, however, that the accumulated number of eligible studies for these two outcomes were all <10, and thus a publication bias test based on a funnel plot and Egger test was not performed.

## Discussion

Published studies revealed that more than 40% of cancer patients who were instructed to receive chemoradiotherapy suffered from oral mucositis (35). As a morbid condition, oral mucositis can lead to cancer patients experiencing several discomforts, such as pain, physical limitations, and psychological distress (4). Moreover, oral mucositis also reduced nutritional intake, weakened mouth care, and decreased the QoL of cancer patients undergoing chemotherapy, radiotherapy, or combination therapy (5). More importantly, oral mucositis was associated with an increase in medical expenditure because it can cause prolonged length of hospitalization and reduced QoL (4, 6). Although several treatment programs have been developed to prevent the occurrence of oral mucositis or decrease complications of oral mucositis among cancer patients undergoing chemotherapy, radiotherapy, or concurrent chemo-radiotherapy (6, 7), the efficacy and safety of these interventions have not been completely confirmed (6). Thus, it is essential to explore further the role of other promising alternatives in preventing and treating oral mucositis induced by chemotherapy, radiotherapy, or concurrent chemoradiotherapy.

This is the first systematic review and meta-analysis to investigate comparative efficacy and safety of oral zinc sulfate in the prevention of chemotherapy-induced oral mucositis. Our meta-analysis found that oral zinc sulfate failed to significantly reduce the incidence of oral mucositis (RR = 0.52, 95% CI = 0.17–1.64) as well as relieve oral mucositits grade (RR = 0.62, 95% CI = 0.11–3.56; RR = 0.70, 95% CI = 0.29–1.71) in cancer patients receiving chemotherapy. Moreover, oral pain intensity, onset of oral mucositis, zinc-related AEs and the QoL between the zinc sulfate and control groups were not significantly different.

Oral mucositis is a condition that appears as a significant result of a series of inflammatory changes in epithelial and subepithelial cells of the oral mucosa because of cytotoxic effects of chemotherapy or radiotherapy (1, 2). Published evidence demonstrated that zinc performs as an organelle stabilizer and a stabilizer of the structure of DNA, RNA, and the ribosome (36). Moreover, some studies also found that zinc is a significant cofactor for DNA synthesis, an important factor for wound healing, and a necessary trace component for the improvement of the immune system (29). Thus, it is speculated that zinc will be of benefit to prevent and treat oral mucositis. However, this study found that the incidence and severity of oral mucositis in the oral zinc sulfate group were not superior to those in the control group. Although the conclusion from Mansouri et al. (12) was in line with our study, four other studies (6, 10, 11, 13) obtained conflicting results. It is to be noted that, in the study performed by Mansouri et al. (12), all eligible patients were assigned to receive high-dose chemotherapy; however, patients in the other four studies were treated with normal dose chemotherapy, and the difference in the dose may be a contributor to the inconsistency in results. In our study, however, we incorporated these studies with high and normal doses into meta-analysis, thus we generated negative findings. Moreover, two previous systematic reviews (14, 37) summarized evidence of adjunctive treatments including zinc sulfate for the prevention and treatment of oral mucositis during cancer therapy and concluded that zinc has positive effects on oral mucositis. However, these two reviews failed to analyze separately the role of zinc in patients receiving a single anticancer treatment. Meanwhile, two recent studies (6, 11) were not included in these two reviews because the latest search was completed in November 2016 (14). These limitations impaired the reliability and robustness of all findings from these two reviews. So, we suggest designing a large-scale study with a similar chemotherapy regime to explore further the potential of zinc sulfate for single anticancer treatment induced oral mucositis, such as chemotherapy-induced oral mucositis.

For the remaining outcomes, we failed to perform quantitative synthesis owing to the lack of valid data, and so descriptive analysis was used to summarize all information. For oral pain intensity, two studies (10, 11) obtained conflicting results; however, Gholizadeh et al. enrolled a relatively large sample size (140 patients in this study) (11), and so the power of the result in this study was higher than that of Arbabi-kalati et al.'s study, in which 50 patients were enrolled. So, zinc sulfate may have failed to alleviate oral pain intensity. Nevertheless, further well-designed studies with large sample sizes are warranted to establish the potential of oral zinc sulfate. For the onset of oral mucositis, Rambod et al. (6) found that the time of occurrence of oral mucositis in the control group was earlier than that in the zinc sulfate group although no significant difference was detected. Published studies (29, 38), which were performed in other population groups, also found that mucositis developed earlier in the control group compared with the zinc sulfate group. Thus, zinc sulfate may have the potential to prevent chemotherapy-induced oral mucositis. To date, only two studies with a limited sample size considered AE and QoL, and so the results must be cautiously considered. We speculated that zinc sulfate may have the potential to improve the QoL of cancer patients undergoing chemotherapy-induced oral mucositis because a promising trend was detected in preventing and treating this given condition.

Although this meta-analysis accumulated a relatively large number of eligible studies with rigorous methods to obtain relatively reliable findings, some limitations in our study must be acknowledged. Firstly, we failed to perform subgroup analysis according to the dose of chemotherapy because of the insufficient number of eligible studies. Thus, further studies should consider the efficacy and safety of different doses of chemotherapy. Secondly, we failed to design a subgroup to investigate the impact of treatment duration on efficacy and safety because sufficient information could not be obtained. Thirdly, the dose of oral zinc used in each study was different; however, we failed to perform subgroup analysis to explore the potential performance of different doses of oral zinc sulfate for the treatment of oral mucositis because a limited number of eligible studies was captured. Finally, all eligible studies included in this study were performed in Iran, and, thus, the findings of our study must be cautiously interpreted when patients from other regions are enrolled.

## Conclusions

We concluded that oral zinc sulfate may not have any clinical benefits in prevention or reduction of incidence, severity, and pain intensity of chemotherapy-induced oral mucositis in cancer patients based on limited data. Nevertheless, it is essential to design RCTs with large-scale and rigorous methods to establish further the efficacy and safety of oral zinc sulfate for chemotherapy-induced oral mucositis before making recommendations owing to the presence of limitations.

## Author contributions

XT and W-QC conceived and designed the study. XT, Y-PP, X-LL, and HC participated in study selection and data extraction. XT, Y-PP, and X-LL performed statistical analysis. XT and HC were involved in manuscript drafting and revision. All authors approved the final manuscript for submission and publication.

### Conflict of interest statement

The authors declare that the research was conducted in the absence of any commercial or financial relationships that could be construed as a potential conflict of interest.
