# Supplementary material for: Oral Zinc Sulfate for Prevention and Treatment of Chemotherapy-Induced Oral Mucositis: A Meta-Analysis of Five Randomized Controlled Trials
Source: Front Oncol. 2018 Nov 19;8:484. doi: 10.3389/fonc.2018.00484 (PMC6252385; doi:10.3389/fonc.2018.00484)
Supplement: Supplementary file 1 [file Table_1.DOCX]

**Search strings for targeted databases**

***PubMed search algorithm***

| **Search** | **Query** |
| --- | --- |
| #8 | Search (((("Zinc Sulfate"[Mesh]) OR ((((((zinc Sulfate[Title/Abstract]) OR solvazinc[Title/Abstract]) OR verazinc[Title/Abstract]) OR zinc sulphate[Title/Abstract]) OR zincomed[Title/Abstract]) OR zincteral[Title/Abstract]))) AND (((("Mucositis"[Mesh]) OR "Stomatitis"[Mesh])) OR ((((((((((Mucositides[Title/Abstract]) OR mucosa irritation[Title/Abstract]) OR mucositis[Title/Abstract]) OR mucosa inflammation[Title/Abstract]) OR Stomatitis[Title/Abstract]) OR Stomatitides[Title/Abstract]) OR Oral Mucositis[Title/Abstract]) OR Oral Mucositides[Title/Abstract]) OR Oromucositis[Title/Abstract]) OR Oromucositides[Title/Abstract]))) AND random* |
| #7 | Search random* |
| #6 | Search ((("Mucositis"[Mesh]) OR "Stomatitis"[Mesh])) OR ((((((((((Mucositides[Title/Abstract]) OR mucosa irritation[Title/Abstract]) OR mucositis[Title/Abstract]) OR mucosa inflammation[Title/Abstract]) OR Stomatitis[Title/Abstract]) OR Stomatitides[Title/Abstract]) OR Oral Mucositis[Title/Abstract]) OR Oral Mucositides[Title/Abstract]) OR Oromucositis[Title/Abstract]) OR Oromucositides[Title/Abstract]) |
| #5 | Search (((((((((Mucositides[Title/Abstract]) OR mucosa irritation[Title/Abstract]) OR mucositis[Title/Abstract]) OR mucosa inflammation[Title/Abstract]) OR Stomatitis[Title/Abstract]) OR Stomatitides[Title/Abstract]) OR Oral Mucositis[Title/Abstract]) OR Oral Mucositides[Title/Abstract]) OR Oromucositis[Title/Abstract]) OR Oromucositides[Title/Abstract] |
| #4 | Search ("Mucositis"[Mesh]) OR "Stomatitis"[Mesh] |
| #3 | Search ("Zinc Sulfate"[Mesh]) OR ((((((zinc Sulfate[Title/Abstract]) OR solvazinc[Title/Abstract]) OR verazinc[Title/Abstract]) OR zinc sulphate[Title/Abstract]) OR zincomed[Title/Abstract]) OR zincteral[Title/Abstract]) |
| #2 | Search (((((zinc Sulfate[Title/Abstract]) OR solvazinc[Title/Abstract]) OR verazinc[Title/Abstract]) OR zinc sulphate[Title/Abstract]) OR zincomed[Title/Abstract]) OR zincteral[Title/Abstract] |
| #1 | Search "Zinc Sulfate"[Mesh] |

***EMBASE search algorithm***

| **No.** | **Query** |
| --- | --- |
| #8 | #3 AND #6 AND #7 |
| #7 | random* |
| #6 | #4 OR #5 |
| #5 | 'mucositis':ti,ab,kw OR 'mucositides':ti,ab,kw OR 'mucosa irritation':ti,ab,kw OR 'mucosa inflammation':ti,ab,kw OR 'oral mucositis':ti,ab,kw OR 'stomatitides':ti,ab,kw OR 'stomatitis':ti,ab,kw OR 'oral mucositides':ti,ab,kw OR 'oromucositis':ti,ab,kw OR 'oromucositides':ti,ab,kw |
| #4 | 'oral mucositis'/exp OR 'mucosa inflammation'/exp OR 'stomatitis'/exp |
| #3 | #1 OR #2 |
| #2 | 'zinc sulfate':ti,ab,kw OR 'solvazinc':ti,ab,kw OR 'verazinc':ti,ab,kw OR 'zinc sulphate':ti,ab,kw OR 'zincomed':ti,ab,kw OR 'zincteral':ti,ab,kw |
| #1 | 'zinc sulfate'/exp |

***CENTRAL search algorithm***

| **No.** | **Query** |
| --- | --- |
| #1 | solvazinc:ti,ab,kw or verazinc:ti,ab,kw or zinc sulphate:ti,ab,kw or zincomed:ti,ab,kw or zincteral:ti,ab,kw in Trials (Word variations have been searched) |
| #2 | MeSH descriptor: [Zinc Sulfate] explode all trees |
| #3 | #1 or #2 |
| #4 | Mucositides:ti,ab,kw or mucosa irritation:ti,ab,kw or mucositis:ti,ab,kw or mucosa inflammation:ti,ab,kw or Stomatitis:ti,ab,kw or stomatitides:ti,ab,kw or Oral Mucositides:ti,ab,kw or oral mucositis:ti,ab,kw or Oromucositis:ti,ab,kw or Oromucositides:ti,ab,kw (Word variations have been searched) |
| #5 | MeSH descriptor: [Mucositis] explode all trees |
| #6 | MeSH descriptor: [Stomatitis] explode all trees |
| #7 | #4 or #5 or #6 |
| #8 | random* (Word variations have been searched) |
| #9 | #3 and #7 and #8 |
