# Supplementary material for: Oral Zinc Sulfate for Prevention and Treatment of Chemotherapy-Induced Oral Mucositis: A Meta-Analysis of Five Randomized Controlled Trials
Source: Front Oncol. 2018 Nov 19;8:484. doi: 10.3389/fonc.2018.00484 (PMC6252385; doi:10.3389/fonc.2018.00484)
Supplement: Supplementary file 2 [file Table_2.DOC]

**Data Extraction Table Designed by Xu Tian**

**Summary of Included** Trial Characteristics

|  | | **Trial 1** | **Trial 2** | **Trial 3** |
| --- | --- | --- | --- | --- |
|  | | | | |
| Design | |  |  |  |
| Duration of follow-up | |  |  |  |
| Location | |  |  |  |
|  | | | | |
| Total number | |  |  |  |
| Age | |  |  |  |
| Sex (male/female) | |  |  |  |
| Baseline characteristics | |  |  |  |
| Assessment of compliance | |  |  |  |
|  | | | | |
| Intervention | |  |  |  |
| Control | |  |  |  |
| Other comparator/s | |  |  |  |
|  | | | | |
| Primary | |  |  |  |
| Secondary | |  |  |  |
| Analysis method | |  |  |  |
| Notes | |  |  |  |
|  | | | | |
| Random sequence generation (selection bias) | Support for judgement |  |  |  |
|  | Author’s judgement | Unclear risk | Low risk | Unclear risk |
| Allocation concealment (selection bias) | Support for judgement |  |  |  |
|  | Author’s judgement | Low risk | Low risk | Unclear risk |
| Blinding (performance bias and detection bias) Investigators and Patients | Support for judgement |  |  |  |
|  | Author’s judgement | Low risk | Low risk | Unclear risk |
| Blinding (performance bias and detection bias) Outcome assessors | Support for judgement |  |  |  |
|  | Author’s judgement | High risk | High risk | Unclear risk |
| Incomplete outcome data (attrition bias) All outcomes | Support for judgement |  |  |  |
|  | Author’s judgement | Low risk | Low risk | Low risk |
| Selective reporting (reporting bias) | Support for judgement |  |  |  |
|  | Author’s judgement | High risk | Low risk | Low risk |
| Other bias | Support for judgement |  |  |  |
|  | Author’s judgement | Low risk | Low risk | Low risk |
